# Airport-FOD3S: A Three-Stage Detection-Driven Framework for Realistic Foreign Object Debris Synthesis

**DOI:** 10.3390/s25154565

**Published:** 2025-07-23

**Authors:** Hanglin Cheng, Yihao Li, Ruiheng Zhang, Weiguang Zhang

**Affiliations:** 1 School of Transportation, Southeast University, Nanjing 211189, China; 2 Luoyang Flight College, Civil Aviation Flight University of China, Luoyang 471132, China

**Keywords:** foreign object debris, image generation, image blending, size transformation, object detection

## Abstract

Traditional Foreign Object Debris (FOD) detection methods face challenges such as difficulties in large-size data acquisition and the ineffective application of detection algorithms with high accuracy. In this paper, image data augmentation was performed using generative adversarial networks and diffusion models, generating images of monitoring areas under different environmental conditions and FOD images of varied types. Additionally, a three-stage image blending method considering size transformation, a seamless process, and style transfer was proposed. The image quality of different blending methods was quantitatively evaluated using metrics such as structural similarity index and peak signal-to-noise ratio, as well as Depthanything. Finally, object detection models with a similarity distance strategy (SimD), including Faster R-CNN, YOLOv8, and YOLOv11, were tested on the dataset. The experimental results demonstrated that realistic FOD data were effectively generated. The Structural Similarity Index Measure (SSIM) and Peak Signal-to-Noise Ratio (PSNR) of the synthesized image by the proposed three-stage image blending method outperformed the other methods, reaching 0.99 and 45 dB. YOLOv11 with SimD trained on the augmented dataset achieved the mAP of 86.95%. Based on the results, it could be concluded that both data augmentation and SimD significantly improved the accuracy of FOD detection.

## 1. Introduction

Foreign Object Debris (FOD) refers to objects that appear on the airport’s runways, taxiways and aprons, and which may cause damage to the aircraft, such as screws, nuts, rubber blocks, and stones [1]. The presence of FOD may pose huge potential risk to aircraft during takeoff and landing, and it needs to be removed from the runway immediately, when it is observed [2]. The traditional FOD detection and removal methods were conducted manually to check runways and other regions at regular time intervals [3,4,5]. Such methods were low in cost and inefficient, and might also miss FOD which was of a small size or of a color that was similar to the runway pavement, such as metal [6].

In addition to manual detection, another technology that utilized automatic detection equipment was also adopted, and could be divided into two types: wave-based methods, which included infrared and millimeter-wave radar, and vision-based methods, which incorporated video and images [7,8,9,10]. The two methods were typically utilized together: the radar was applied to scan the runway and find potential FOD, and the images were further used to ensure if FOD really existed [11]. With the development of optical detection equipment and the wide application of artificial intelligence technology, many FOD detection methods based on deep learning and computer vision were proposed [12,13,14,15,16]. However, in the applications of FOD detection, this technique still faced two problems: (a) images of FOD did not match the actual scene, and (b) the image did not reflect the true size of the FOD.

Due to the high safety requirements of the airport work area, FOD image acquisition under ideal conditions could not be carried out on the real runway. Currently, most FOD images are taken by randomly placing FOD on the road surface, and the amount of FOD of various categories was unbalanced. Furthermore, in the field site, FOD was too small to be easily recognized, since it was very small in size compared to the surrounding concrete slab, whereas the FOD collected from the road surface was typically well designed and large in size, which made it easy to detect it [17,18,19,20]. The lack of large amounts of accurately labeled data was a significant challenge in applying deep learning detection algorithms to FOD detection. However, the unique nature of FOD detection—such as the variability in object types, sizes, and environmental conditions—made it difficult to collect and label sufficient data for training deep learning models effectively [21,22,23,24].

In addition to existing airport-specific datasets, recent developments in road scene understanding have introduced large-scale benchmarks that are also relevant for object detection in safety-critical environments. For example, the RSUD20K dataset [25] provides 20,000 high-resolution images with diverse annotations of vehicles, pedestrians, road signs, and rare objects, under various weather and lighting conditions. While RSUD20K is primarily designed for autonomous driving, its diverse environmental coverage and emphasis on small, irregularly shaped objects make it conceptually relevant to FOD detection tasks in airport environments. Therefore, it was necessary to expand the FOD data to establish a comprehensive dataset.

Data augmentation methods using existing data were categorized into supervised and unsupervised methods [26]. Supervised data augmentation techniques such as geometric transformations, color transformations, and multi-sample data blending were employed initially to enhance the airport FOD data, with corresponding adjustments made to the labels [27]. Unsupervised data augmentation methods, such as Generative Adversarial Networks (GANs) and diffusion models, were further used to expand the airport FOD data across different scenarios [28]. The limitation of supervised data augmentation lay in its reliance on precise label adjustments and its difficulty in generating new samples beyond the original data distribution, which restricted the enhancement of data diversity and generalization capability [29].

The unsupervised data augmentation methods leveraged generative models to synthesize new image data. Although these methods hold potential for expanding datasets, their performance in generating FOD images for airport scenarios was suboptimal, primarily due to the difficulty in accurately capturing the intricate details of complex environments and small objects, as well as the significant domain gap between generated and real data [30]. Meanwhile, insufficient training data, inherent limitations of generative models, and imprecise annotation information further constrain the quality and practicality of the generated images [31].

The conditionally controlled image blending methods demonstrated significant potential in augmenting airport FOD data, and were capable of more accurately simulating the distribution of FOD in real-world scenarios, while addressing the issue of mismatches between generated images and actual environments. Direct blending led to significant discrepancies in FOD size, which critically influenced the judgment of FOD risk levels, underscoring the importance of accurate size correspondence of objects in the synthesized image [32]. To make the synthesized image appear more natural, the blending boundary should be seamless. However, if there were significantly different texture features between the source and target images, the directly synthesized image would have obvious boundaries. When there was a substantial difference in the styles of the two images, the synthesized image would also appear incompatible [33]. Therefore, when performing image blending, it was essential to consider the image boundary, style, and size correspondence.

The current deep learning detection algorithms exhibited suboptimal performance in the detection of small-target FOD [34]. In order to better detect FOD with random locations and various shapes, modules such as the attention mechanism and multi-scale feature extraction and blending had been proposed [35,36,37]. The distribution of FOD in different locations should be considered, especially the fact that some FOD locations were varying over time and cannot be easily calculated. The output of the current detection algorithm was the bounding box of the target in the image, which could not provide the real position and size of the target, and could not guide the staff to process quickly and accurately [38].

**Figure 1:**
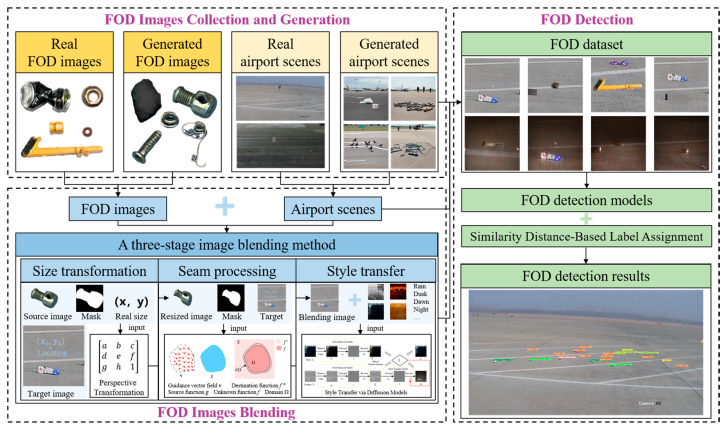
Overall framework of the proposed Airport-FOD3S method. The framework consists of three major modules: (1) data generation and augmentation of FOD and airport scenes under varied conditions, (2) a three-stage image blending process including size transformation, seam processing, and style transfer, and (3) evaluation through object detection models and image quality metrics.

The proposed framework, named Airport-FOD3S, integrates three critical stages for realistic FOD synthesis: size transformation to ensure accurate consistency of object scaling, seamless processing to eliminate blending artifacts, and style transfer to harmonize synthetic objects with environmental conditions. The framework was divided into three main parts: FOD image collection and generation, FOD image blending, and FOD detection, as shown in Figure 1. First, the placement and pitch angle of cameras in airport FOD monitoring scenarios were determined, and images of key monitoring areas and FOD were acquired. Image data augmentation was performed using GANs and diffusion models, generating images of monitoring areas under different environmental conditions and a rich variety of FOD images. Second, a three-stage image blending method that integrated size transformation, seam processing, and style transfer was proposed to enhance the realism and consistency of FOD image blending. A multi-reference point perspective transformation matrix was constructed, and precise size adjustments are applied to the source images based on the blending location. An improved seam processing technique was adopted, which combines gradient blending and multi-resolution blending to effectively eliminate seam artifacts. Additionally, style transfer techniques were introduced to enhance the uniformity of the synthetic images. And the image quality of different blending methods was quantitatively evaluated using metrics such as Structural Similarity Index (SSIM) and Peak Signal-to-Noise Ratio (PSNR). Finally, the performance of various object detection models was compared on the augmented dataset and employed a Similarity Distance Strategy (SimD) to enhance the detection accuracy of small target FOD. This comprehensive process effectively improved the intelligent detection accuracy of small target FOD in real airport environments.

## 2. Methodology

### 2.1. Data Augmentation

#### 2.1.1. Airport and FOD Generation Based on Diffusion

**Figure 2:**
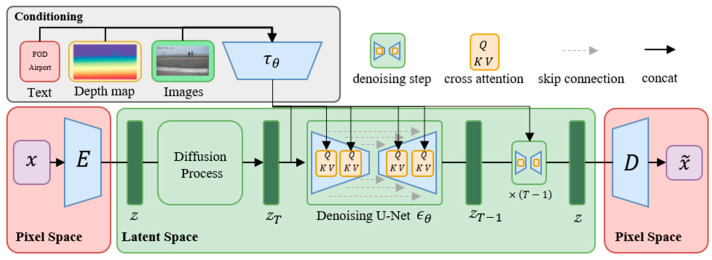
Architecture of the FOD Stable Diffusion model used for generating synthetic FOD images.

FOD stable diffusion is utilized to generate high-quality images of FOD in airports and its structure is shown in Figure 2 [39]. The Latent Diffusion Model (LDM) establishes a three-phase framework for efficient and controllable image synthesis. First, a perceptual compression encoder projects images into a low-dimensional latent space, where adversarial losses and Kullback–Leibler/Vector Quantization regularization jointly eliminate high-frequency artifacts such as sensor noise or background interference while preserving semantic fidelity. The decoder reconstructs the images put through with minimal distortion. Subsequently, a diffusion process is optimized in this latent space by training a time-conditioned UNet denoising network $\epsilon_{\theta }$ using the objective, which reduces computational overhead by orders of magnitude compared to pixel-space diffusion while maintaining sensitivity to localized anomalous features of microscale artifacts through convolutional inductive biases, as shown in Equation (1).

(1) $$\;L_{LDM}=E_{\varepsilon \left(x\right),\epsilon \sim N\left(0,1\right),t}\left[{\left\|\epsilon -\epsilon_{\theta }\left(z_{t},t,\tau_{\theta }\left(y\right)\right)\right\|}_{2}^{2}\right].$$

Here, $\epsilon_{\theta }$ is a time-conditional UNet that denoises the latent $z_{t}$ at diffusion step t, $while\;\tau_{\theta }\left(y\right)$ is the conditioning encoder of input prompt y.

Finally, a multimodal conditioning mechanism integrates external guidance such as text, depth maps and images via cross-attention layers, enabling dynamic alignment between latent representations and conditional inputs.

(2) $$\;\mathrm{Attention}(Q,K,V)=\mathrm{softmax}(\frac{QK^{T}}{\sqrt{d}})\cdot V,\;Q=W_{Q}^{\left(i\right)}\cdot \varphi_{i}\left(z_{t}\right),\;K=W_{K}^{\left(i\right)}\cdot \tau_{\theta }\left(y\right),\;V=W_{V}^{\left(i\right)}\cdot \tau_{\theta }\left(y\right).$$

Here, $\varphi_{i}\left(z_{t}\right)\in R^{N\times d_{\epsilon }^{i}},$ an intermediate representation of the UNet implementing $\epsilon_{\theta }$ and $W_{Q}^{\left(i\right)}\in R^{d\times d_{\tau }}$, $W_{K}^{\left(i\right)}\in R^{d\times d_{\tau }}$, $W_{V}^{\left(i\right)}\in R^{d\times d_{\epsilon }^{i}}$ are learnable projection matrices.

This architecture allows precise control over spatial layouts and attributes of synthesized content under complex interference scenarios, ensuring robustness to spatially heterogeneous perturbations through semantic-aware feature modulation.

#### 2.1.2. Airport Scene Generation in Different Condition

**Figure 3:**
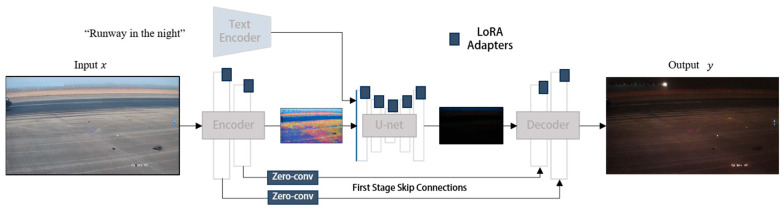
Enhanced generator architecture based on CycleGAN-Turbo for airport scene translation.

The generator architecture from CycleGAN-Turbo is adopted to rapidly generate airport scene images under different lighting and weather conditions [40]. This architecture is based on an LDM and integrates three independent modules into an end-to-end neural network, with only a small number of additional trainable weights, as shown in Figure 3. These three modules are the Low-Rank Adaptation (LoRA) adapters, skip connections and Zero-Convs, and retraining of the first layer of the U-Net, which enhance the conditional control capability of the pre-trained LDM while maintaining fast inference speed.

First, LoRA adapters are introduced into multiple layers of the U-Net, leveraging low-rank adaptation to fine-tune the pre-trained diffusion model for new image translation tasks without requiring large-scale retraining of the entire model. Second, skip connections and Zero-Convs are employed to preserve scene structure and reduce distortion. Skip connections establish a bridge between the input image and the output image, ensuring structural consistency, while Zero-Convs enable effective conditioning input control over the diffusion process with minimal perturbation to the pre-trained model. Finally, the first layer of the U-Net is retrained to better adapt the model to new task domains and mitigate conflicts between the input image and the diffusion process. These modules are seamlessly integrated into the LDM framework, forming an end-to-end architecture that supports various image-to-image translation tasks while adding only a small number of additional parameters. By combining LoRA fine-tuning, structural preservation mechanisms, and selective retraining, our method effectively enhances the pre-trained diffusion model, achieving high-quality image translation while maintaining efficient inference speed.

### 2.2. A Three-Stage Image Blending Approach Considering Size, Seam, and Style

Considering that the actual size and pixel dimensions of the FOD source images are known, and that the transformation matrix between the target image and the real-world scene is determined once the acquisition setup is fixed, a pixel-scale transformation of the source image is performed, based on its specific placement within the target image. This ensures size alignment between the source and target images under random positioning conditions. Therefore, this section proposes a three-stage image blending algorithm that accounts for object size and scene mapping. First, the source and target images are aligned in terms of actual dimensions. During the transformation process, the mask of the source image is adjusted in both size and position, according to the random placement. Finally, seamless image blending and style transformation operations are applied.

#### 2.2.1. Size Transformation of Source Images

In the process of image blending, accurately representing the size of FOD is crucial. To achieve this, the precise image masks are created to isolate FOD from its backgrounds. Within these masks, dimensioning is performed to measure each object’s size accurately. This approach ensures that the FOD size was correctly represented in the synthetic images, contributing to realistic size transformation and enhanced accuracy in object detection.

**Figure 4:**
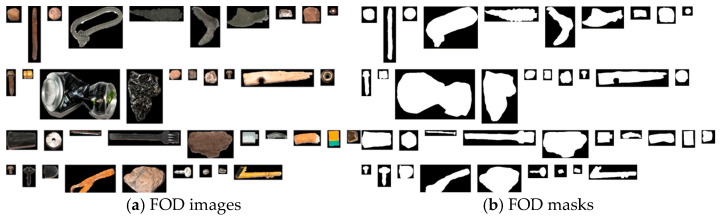
FOD image collection in real airport scenarios.

Various types of FOD are systematically collected in both simulated and real airport environments, followed by a meticulous process of data augmentation and synthesis. This undertaking aims to curate a substantial and evenly distributed FOD dataset. Figure 4 illustrates selected instances of these FOD items, characterized by their diverse attributes, including color, shape, material composition, and size, spanning a dimensional range from 1 to 30 cm. This dataset encompasses minuscule FOD, such as nuts, bolts, diminutive aircrew tool components, tire fragments, and pieces of pavement, as shown in Figure 4.

By precisely aligning the known physical dimensions of the FOD with its expected spatial distribution in the airport scene, the transformation matrix is calibrated to ensure that scale, orientation, and perspective distortion are accurately modeled, effectively mapping the object dimensions from the source image into the target scene’s coordinate system. A spatial mapping function, constructed based on the actual dimensions of the FOD, leverages a multi-reference point transformation technique to compensate for variations in camera viewpoints, depth, and lens distortions inherent to the airport scene. Meanwhile, to address the complex distortions induced by perspective foreshortening and depth variations, a nonlinear deformation technique is employed, which adjusts the object contours while preserving its relative geometric proportions, thereby ensuring that the object maintains a natural appearance, even under severe perspective changes.

**Figure 5:**
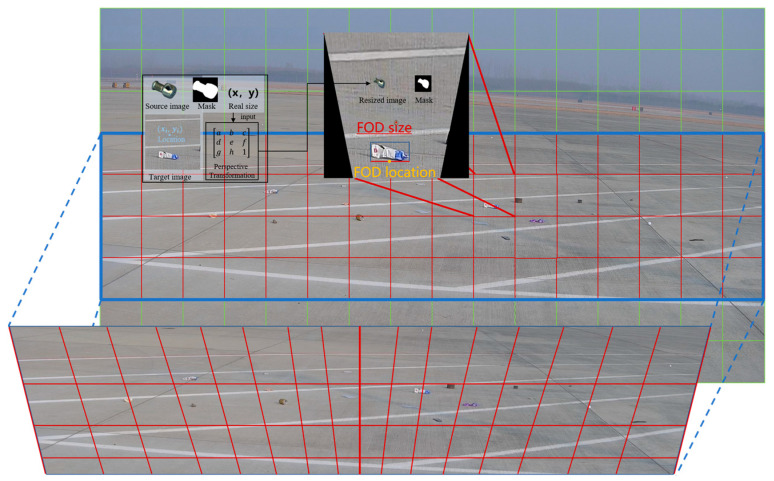
Computation of the image block transformation matrix for size alignment.

FOD at the airport typically appears in a fallen state, which means its height is generally the smallest dimension. When addressing the problem of depth and size estimation in monocular images for FOD detection from a wide-angle perspective, the three-dimensional problem is simplified into a two-dimensional one. The longest dimension of the object is approximated as the length of its bottom edge in the image, and its position is simplified as the midpoint of this bottom edge. In real-world scenarios, FOD predominantly appears within the red-marked areas. Therefore, when performing size transformation, the conversion is mainly focused on the red-marked regions, as shown in Figure 5. The red-marked area is divided into 4 × 16 small square blocks, each with a side length of 240 pixels. Based on the camera placement and tilt angle, the perspective transformation matrix for each of these 64 small square blocks relative to the actual scene is calculated. Using the blending position ($x_{i},y_{i})$, the corresponding perspective transformation matrix for the designated region is determined. By inputting the source image and its mask, along with the size of the FOD, a projection transformation is applied to align it with the actual scene, resulting in the size-adjusted source image and its corresponding mask.

To align the FOD object with the spatial geometry of the airport scene, a perspective transformation matrix $H\in R^{3\times 3}$ is estimated to map the source image coordinates to a selected planar region in the target image:

(3) $$\;x_{t}∼H\cdot x_{s}.$$

where $x_{s}=[x_{s},y_{s},1{]}^{T}$ and $x_{t}=[x_{t},y_{t},1{]}^{T}$ are homogeneous coordinates in the source and target domains, respectively.

The matrix H is computed by solving a planar homography using four or more corresponding reference points between the source mask contour and the designated target region. These target points are pre-defined anchors located within each scene block, calibrated based on simulated camera geometry and scene depth. The optimal homography is obtained by minimizing the reprojection error:

(4) $$\;\underset{H}{min}\sum_{i=1}^{N}{∥p_{i}^{\prime }-\frac{H\cdot p_{i}}{h_{3}^{T}\cdot p_{i}}∥}^{2}.$$

Once H is estimated, it is applied to both the FOD image and its binary mask, preserving pixel-wise alignment and spatial scaling.

Let $l_{real}\in R$ denote the real-world length of the FOD, and $r_{scene}$. be the estimated spatial resolution at the projected region of the target image. The expected pixel length $l_{pixel}$ in the image is then

(5) $$\;l_{pixel}=l_{real}\cdot r_{scene}.$$

The source image $I_{FOD}$ and its corresponding binary mask M are scaled by a factor s to achieve this length:

(6) $$\;s=\frac{l_{pixel}}{l_{src}}.$$

where $l_{src}$ is the original pixel length of the FOD in the source image. After computing s, we obtain

(7) $$\;I_{FOD}^{rescaled}=Resize(I_{FOD},s),$$

(8) $$M^{rescaled}=Resize(M,s).$$

To address the geometric distortions induced by perspective compression, lens curvature, and depth differences, a nonlinear deformation field D(x,y) is introduced. The final transformed coordinates of each pixel are

(9) $$\;x_{t}^{\prime }=H\cdot x_{s}+D(x,y).$$

where $D(x,y)=[\delta_{x}(x,y),\delta_{y}(x,y){]}^{T}$ is a smooth displacement field learned or interpolated using thin-plate splines or grid-based control points, preserving continuity along mask boundaries.

The final transformed binary mask is denoted as

(10) $$M^{\prime }=W\left(M^{rescaled},H,D\right).$$

where W denotes the joint warp function combining both affine and non-affine transformations. This transformed mask $M^{\prime }$ guides the seamless image blending and local style adaptation processes.

#### 2.2.2. Seam Processing

To eliminate visible seams between the synthetic images, an improved seam processing technique is implemented [41]. This technique combines gradient blending and multi-resolution blending methods, where gradient blending smooths the transitions between images, and multi-resolution blending integrates image details at different scales to eliminate artifacts.

The Poisson blending method formalizes the image blending as an image interpolation problem using the guiding vector field, as shown in Equation (3).

(11) $$\;\underset{f}{min}\iint_{\Omega }|\nabla f-v{|}^{2}\;\mathrm{with}\;{\left.f\right|}_{\partial \Omega }={\left.f^{*}\right|}_{\partial \Omega }.$$

where ∇ is the Laplacian gradient operator, f is the function of the synthetic image, $f^{*}$ is the function of the target image, v is the vector field, Ω is the blending region, and ∂Ω is the boundary of the blending region. In this case, the guiding field v is the gradient field directly obtained from the source image g, v= ∇g.

The minimization problem with boundary conditions is solved independently for each color channel of the RGB image. The problem is discretized using the underlying discrete pixel grid, thereby transforming it into a quadratic optimization problem; that is, for all pixel points in the blending region, under the condition that the function values of the target image and the synthetic image are equal, the pixel change at the blending boundary is minimized.

(12) $$\;\underset{{\left.f\right|}_{\Omega }}{min}\sum_{\left\langle p,q\cap \Omega \neq \emptyset \right\rangle }{\left(f_{p}-f_{q}-v_{pq}\right)}^{2},\;\mathrm{with}\;f_{p}=f_{p}^{*}\;\mathrm{for}\;\mathrm{all}\;p\in \partial \Omega .$$

where $N_{p}$ is the set of the four connected regions of pixel p, and $\left\langle p,q\right\rangle$ represents the pixel pair. For all $\left\langle p,q\right\rangle$ within the connected region, q is the pixel point of the connected region of pixel p, and $f_{p}$ is the function value at pixel p, $v_{pq}=g_{p}-g_{q}$.

For the discrete system of the image pixel grid, the solution of the problem usually needs to consider the relationship between discrete data points, and the solution can be transformed into the following linear equations.

For the pixel point p within the blending region Ω, the linear equations are as in Equation (5).

(13) $$\;\left|N_{p}\right|f_{p}-\sum_{q\in N_{p}\cap \Omega }f_{q}=\sum_{q\in N_{p}\cap \partial \Omega }f_{q}^{*}+\sum_{q\in N_{p}}v_{pq}.$$

For the pixel point p located inside region Ω, there are no boundary pixels from the target image. Therefore, the equation becomes Equation (6).

(14) $$\;\left|N_{p}\right|f_{p}-\sum_{q\in N_{p}}f_{q}=\sum_{q\in N_{p}}v_{pq}.$$

#### 2.2.3. Style Transfer

**Figure 6:**
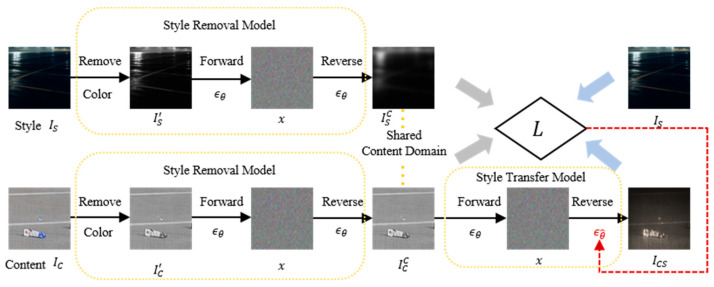
The Architecture of StyleDiffusion.

The StyleDiffusion was used to enhance the synthetic FOD images under different lighting and weather conditions, which is a diffusion-based framework for disentangling and transferring style in images [42]. It consists of three key components: (1) a style removal module, which employs a diffusion model to extract content by eliminating style elements such as color and texture; (2) a style transfer module, which utilizes a pre-trained diffusion model and a CLIP-based loss function to learn and transfer disentangled style information; and (3) a CLIP-based style disentanglement loss with a prior style reconstruction, ensuring effective style representation and transfer [43]. The framework leverages the diffusion process for both content extraction and style transfer, providing controllability and high-quality stylization, as shown in Figure 6. The style disentanglement loss incorporates both L1 and directional constraints, ensuring alignment between the learned style and the target style domain. Finally, a prior style reconstruction further refines the stylization process.

#### 2.2.4. Evaluation of Image Blending Performance

To comprehensively evaluate the effectiveness of image blending, we adopt a combination of qualitative and quantitative methods. Qualitative analysis primarily relies on visual inspection to assess the perceptual realism and seamless integration of the synthetic images, while quantitative analysis employs Structural Similarity Index Measure (SSIM), Peak Signal-to-Noise Ratio (PSNR), and DepthAnything-based depth consistency analysis to quantify blending quality and spatial consistency [44,45,46].

For qualitative evaluation, we begin with visual inspection to subjectively assess the synthetic images, focusing on key aspects such as perceptual realism, seamless blending, and blending consistency. Perceptual realism refers to whether the synthetic image aligns with real-world visual features and integrates naturally into the scene. Seamless blending evaluates how well the FOD blends with the background without noticeable edge artifacts or color mismatches. Lastly, blending consistency examines the stability of the blending effect under varying lighting, weather conditions, or perspectives, ensuring no abrupt or inconsistent visual effects.

For quantitative evaluation, we use multiple metrics to objectively measure blending performance. SSIM is employed to assess the similarity between the synthetic image and the reference image, focusing on the preservation of luminance, contrast, and structural information. The SSIM is calculated as follows:

(15) $$\;SSIM\left(x,y\right)=\frac{\left(2\mu_{x}\mu_{y}+C_{1}\right)\left(2\sigma_{xy}+C_{2}\right)}{\left(\mu_{x}^{2}+\mu_{y}^{2}+C_{1}\right)\left(\sigma_{x}^{2}+\sigma_{y}^{2}+C_{2}\right)}.$$

where $\mu_{x}$ and $\mu_{y}\;$ are the mean intensities of images x and y, $\sigma_{x}^{2}$ and $\sigma_{y}^{2}$ are their variances, $\sigma_{xy}$ is the covariance, and $C_{1}$ and $C_{2}$ are constants for stability. A higher SSIM value, closer to 1, indicates better structural similarity and blending quality.

PSNR is used to measure the signal strength relative to noise in the synthetic image, serving as an indicator of image fidelity. It is computed as follows:

(16) $$\;PSNR=10\log_{10}\left(\frac{{MAX}_{I}^{2}}{MSE}\right).$$

where ${MAX}_{I}$, the maximum possible pixel value of the image, and the Mean Squared Error (MSE), are calculated as

(17) $$\;MSE=\frac{1}{mn}\sum_{i=0}^{m-1}\sum_{j=0}^{n-1}{\left[I\left(i,j\right)-K\left(i,j\right)\right]}^{2}.$$

where $I\left(i,j\right)$ and $K\left(i,j\right)$ represent the pixel values of the reference and synthetic images, respectively. A higher PSNR value indicates lower distortion and better visual quality.

Additionally, DepthAnything is utilized to estimate depth maps before and after blending and compare the depth relationships between FOD and the surrounding environment. This ensures that the spatial alignment of the synthetic FOD remains realistic, and does not introduce depth inconsistencies or spatial misalignments.

### 2.3. SimD-Based Label Assignment Strategy

**Figure 7:**
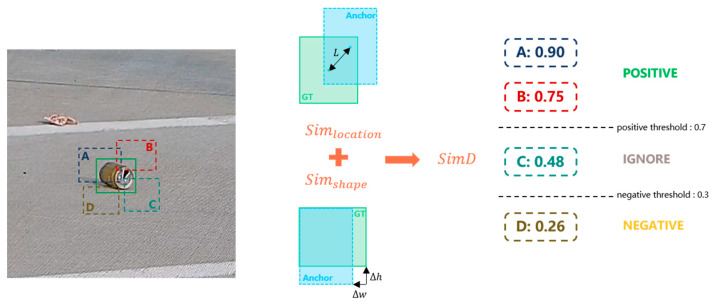
The SimD-based label assignment strategy.

To address the inherent limitations of IoU-based label assignment in detecting small, deformable, or partially occluded FOD, a novel similarity metric termed Similarity-aware Detection Distance (SimD) is proposed. Unlike traditional IoU, which solely measures the geometric overlap between bounding boxes, SimD integrates both spatial alignment and geometric consistency, offering a more robust and perceptually aligned criterion for anchor matching, as shown in Figure 7.

The design motivation behind SimD lies in its ability to preserve semantic coherence between predicted and ground-truth objects, particularly when visual overlap is minimal or unreliable. For instance, in airport scenarios, FOD items often exhibit irregular shapes, small spatial footprints, or ambiguous contours, due to motion blur, low contrast, or environmental occlusions. Under such conditions, IoU can assign disproportionately low scores to otherwise reasonable detections, thereby increasing false negatives and impairing model training. SimD mitigates this by introducing a composite metric that accounts for both positional proximity and shape similarity.

Formally, SimD is defined as a weighted combination of two components: the exponential of the squared distance between the center points of the predicted and ground-truth boxes, and a normalized area similarity term that reflects their geometric alignment. The equation is expressed as

(18) $$\;SimD=e^{-\left({sim}_{location}+{sim}_{shape}\right)},$$

(19) $${sim}_{location}=\sqrt{{\left(\frac{x_{g}-x_{a}}{\frac{1}{m}\times \left(w_{g}+w_{a}\right)}\right)}^{2}+{\left(\frac{y_{g}-y_{a}}{\frac{1}{n}\times \left(h_{g}+h_{a}\right)}\right)}^{2}},$$

(20) $${sim}_{shape}=\sqrt{{\left(\frac{w_{g}-w_{a}}{\frac{1}{m}\times \left(w_{g}+w_{a}\right)}\right)}^{2}+{\left(\frac{\left(h_{g}-h_{a}\right)}{\frac{1}{n}\times \left(h_{g}+h_{a}\right)}\right)}^{2}},$$

(21) $$m=\frac{\sum_{i=1}^{M}\sum_{j=1}^{N_{i}}\sum_{k=1}^{Q_{i}}\frac{\left|x_{ij}-x_{ik}\right|}{w_{ij}+w_{ik}}}{\sum_{i=1}^{M}N_{i}\times Q_{i}},$$

(22) $$n=\frac{\sum_{i=1}^{M}\sum_{j=1}^{N_{i}}\sum_{k=1}^{Q_{i}}\frac{\left|y_{ij}-y_{ik}\right|}{h_{ij}+h_{ik}}}{\sum_{i=1}^{M}N_{i}\times Q_{i}}.$$

where $\left(x_{g},y_{g}\right)$ and $\left(x_{a},y_{a}\right)$ represent the center coordinate of ground truth and anchor, and $w_{g}$, $\;w_{a}$, $\;h_{g}$, $\;h_{a}$ represent the width and height of ground truth. m is the average ratio of the distance in the x-direction to the sum of the two widths for all ground truths and anchors in each image in the whole train set.  M represents the number of images in the train set, and $N_{i}$, $\;Q_{i}$ represent the number of ground truths and anchors in the i-th image. $x_{ij}$, $\;x_{ik}$ represent the x-coordinate of the center point of j-th ground truth and k-th anchor in the i-th image. $w_{ij}$, $\;w_{ik}$ represent the width of j-th ground truth and k-th anchor in the i-th image.

This formulation offers several key advantages. First, the spatial term ensures that predictions located near the actual object receive meaningful similarity scores, even in the absence of substantial overlap. Second, the geometric term penalizes size mismatches and promotes aspect ratio consistency, which is particularly relevant for elongated or irregularly shaped debris. Finally, the smooth and differentiable nature of the SimD metric supports stable gradient propagation during training.

## 3. Experiment

### 3.1. Data Collection

#### Field Data Collection

**Figure 8:**
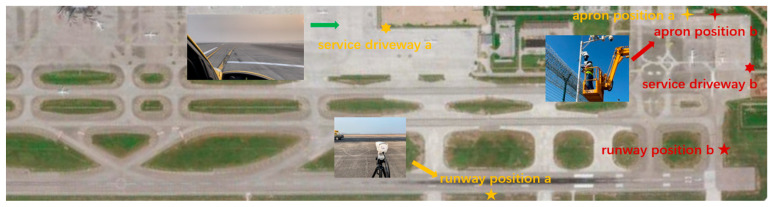
FOD image collection in real airport scenarios.

As shown in Figure 8, cameras were positioned at key monitoring areas of the airport runway with fixed locations and pitch angles to capture a wide field of view, ensuring coverage of both the near and far ends, while accounting for perspective distortion. Images were collected under diverse environmental conditions, including various weather and lighting scenarios, to enhance dataset diversity. FOD was initially placed in customary locations such as aprons, runways, and taxiways and systematically captured in real airport settings during both daytime and night-time under clear weather conditions. To better simulate real-world scenarios, the FOD was then randomly dispersed at different locations and orientations on the runway, replicating its dynamic and unpredictable occurrence.

Further image acquisition of FOD occurred on a broader expanse resembling an airport runway, encompassing various meteorological conditions, including daytime and night-time rain and fog. The image acquisition process was also replicated under two distinct configurations: one involving regular placements and the other involving random placements.

### 3.2. Data Augmentation

Diffusion models were utilized to generate different types of FOD samples (such as metal, plastic, and rubber) and airport scene images (including aprons, runways, and taxiways). The images were further processed via GAN-based models to perform daytime-to-night-time scene conversion, resulting in a comprehensive dataset of airport scene images containing FOD under diverse and realistic environmental conditions. This paper proposed a three-stage blending method, referred to as the deep blending method. The generated and real FOD images were fused with generated and real airport scenes using blending techniques such as copy-paste, Poisson blending, deep blending, and their variants, integrated with size transformation. Image blending was performed using airport scene images captured during daytime and night-time conditions, along with FOD images obtained previously. Both scene images and FOD images included field-collected images and virtually generated images. The blending methods employed were copy-paste, Poisson blending, deep blending, size transformation + copy-paste, size transformation + Poisson blending, and size transformation + deep blending.

### 3.3. FOD Detection

The detectors such as Faster R-CNN, YOLOv8, and YOLOv11 were implemented [47,48,49,50]. The training parameters were setting as Adam optimizer (learning rate = 1 × 10^−4^, weight decay = 5 × 10^−4^), batch size = 64, epochs = 1000, and early stopping (patience = 100 epochs). The ablation experiments were conducted on SimD and the datasets with and without augmentation. The training was conducted in the PyTorch2.0 framework on a computer equipped with an Intel Inter(R) Xeon 6226R CPU, 128 GB of DDR5 memory, and a 24 GB NVIDIA 4090 GPU with CUDA11.3., which from Dell (China) Co., Ltd., Beijing, China.

## 4. Results and Discussion

### 4.1. Generation of FOD Images at Airport

**Figure 9:**
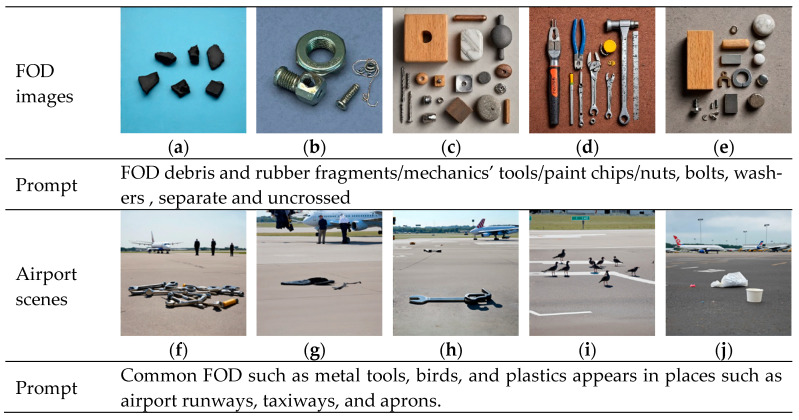
FOD and airport images generated by FOD stable diffusion. (**a**) Rubber; (**b**) Nut; (**c**) Wood block; (**d**) Tool; (**e**) Component; (**f**) Metal; (**g**) Plastic; (**h**) Wrench; (**i**) Bird; (**j**) Trash.

Figure 9 presents the images of FOD and airport scenes generated by the diffusion model after input prompts. For the images of FOD, the input prompts mainly consisted of phrase descriptions of different categories. To facilitate the subsequent extraction of FOD masks, the requirement of separating each item of FOD individually was added. Figure 9a showed a generated broken piece of aircraft tire rubber, which closely resembled the tire spalling in a real scenario. Figure 9b displayed common metal nuts, screws, and iron wires. Figure 9c,e depicted metal, wooden, and plastic parts that could potentially appear at the airport. Figure 9d represented a common aircraft maintenance tool. Notably, the objects in the FOD images generated by the generative model all exhibited friction marks, wear, and other forms of damage, which corresponded highly to FOD in the real environment. For airport scenes, the input prompts were mainly scene pictures of airport runways, taxiways, and aprons with common FOD made of materials such as metal and plastic. Figure 9f–j showed the image details output with the added restriction condition of the monitoring orientation beside the runway. In some scene details such as the segmentation of runway pavement, cement and asphalt materials, and bird shadows, the performance was relatively good, and consistent with the real environment. A common problem in these images was that the size of FODs was seriously mismatched with the proportions of backgrounds such as people and airplanes. The FOD appeared too large and was too densely distributed, so could not be directly used as FOD detection data. The diffusion model had been used to generate different kinds of FOD images and hard-to-obtain airport scene images for image blending.

**Figure 10:**
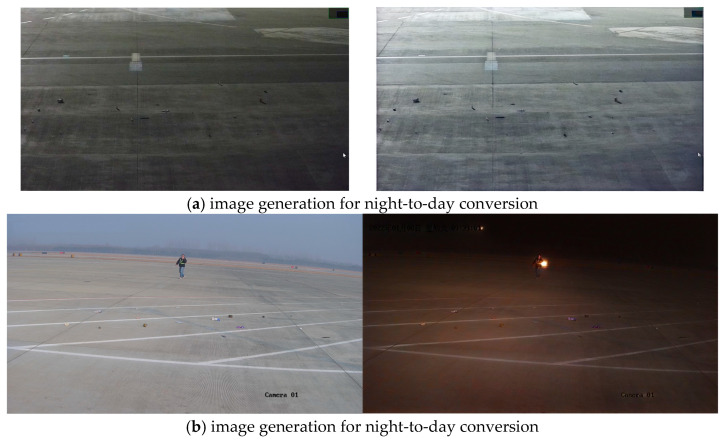
FOD image collection in real airport scenarios.

Since the generated airport scenes could not fully match the objective world, only a small number of them were added for data augmentation. To further enhance data diversity, CycleGAN-Turbo was employed to augment images of real airport scenes under different lighting and environmental conditions, as shown in Figure 10.

### 4.2. Image Blending

#### 4.2.1. Blending Performances in Daytime

**Figure 11:**
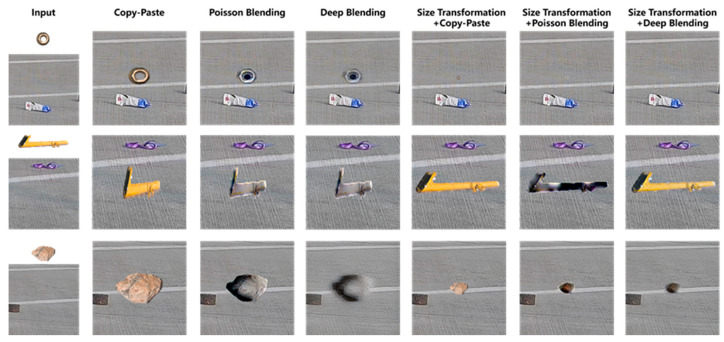
FOD images collection in real airport scenarios.

As illustrated in Figure 11, a comparative analysis of various image blending methods were conducted for integrating FOD (nuts, pipe wrenches, and stones) into daytime airport apron scenes. The experimental results revealed that omitting size transformation during the integration process leads to significant scale ratio imbalances among distinct items of FOD, resulting in perceptually unnatural visual representations. The presence of chromatic interference from extraneous colored objects (e.g., purple lanyards) demonstrated notable limitations in conventional blending techniques. The Poisson blending method exhibited susceptibility to color contamination, as evidenced by the chromatic distortion observed in yellow-wrench instances adjacent to purple elements. Comparative evaluations of different augmentation pipelines demonstrated that the size transformation with deep blending (ST + DB) method achieved superior visual fidelity when reconstructed in real airport environments. It was concluded that, for daytime apron scenarios, the ST + DB approach provides optimal performance in FOD data augmentation, while alternative methods serve complementary roles in enhancing dataset diversity.

**Figure 12:**
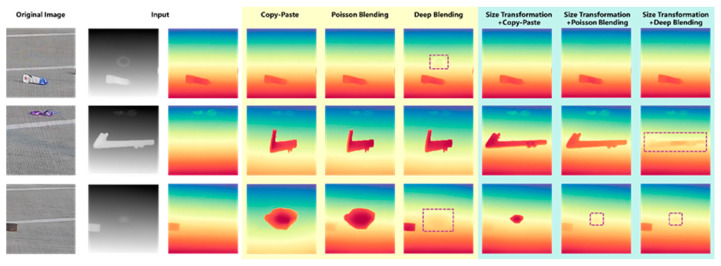
Depth estimation of different blending methods in daytime airport.

To further explore the influence of different blending methods on the blending positions of FOD, the depth estimation maps were plotted of airport scenes under different blending approaches, as shown in Figure 12. It could be seen from the maps that when a nut with a relatively small size was fused with the airport scene in the daytime, neither the direct copy-paste method nor the Poisson blending method could well reflect the positional information of the nut in the airport scene, while the deep blending method could reflect the approximate position of the nut. When undergoing size transformation, due to the small proportion of the nut in the scene, its position failed to be manifested. When a large-sized pipe wrench was fused with the airport scene in the daytime, only the method combining size transformation and deep blending could better reflect the position of the pipe wrench.

**Figure 13:**
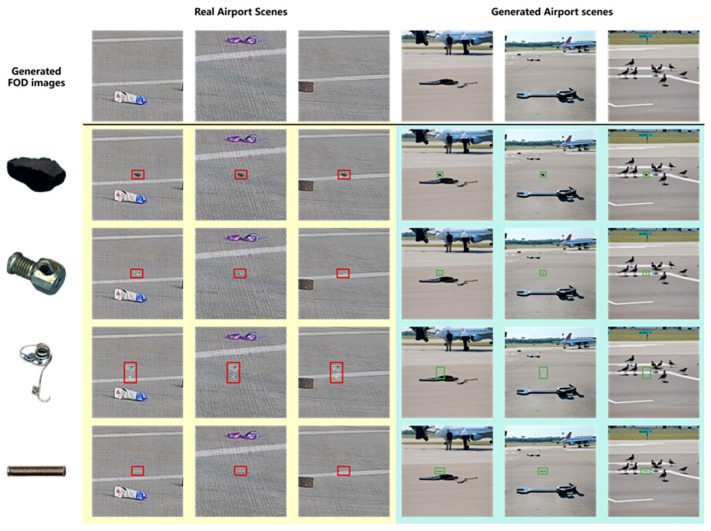
The fusion effect of generated foreign object debris (FOD) images with real/generated airport scenes.

As illustrated in Figure 13, the results of deep blending between the generated FOD images and both real and generated airport scene images were presented. It could be observed that FOD of varying materials and shapes achieved effective integration with airport images. When blended with real apron images featuring prominent groove textures, the edges of the FOD retained the underlying groove patterns. Conversely, when fused with synthesized airport scenes with less distinct pavement textures, the object edges appeared relatively smoother. When FOD was positioned near airport markings, the imaging effects of the markings on the objects were well preserved. These observations were consistent with real-world scenarios of FOD presence in airport environments.

**Figure 14:**
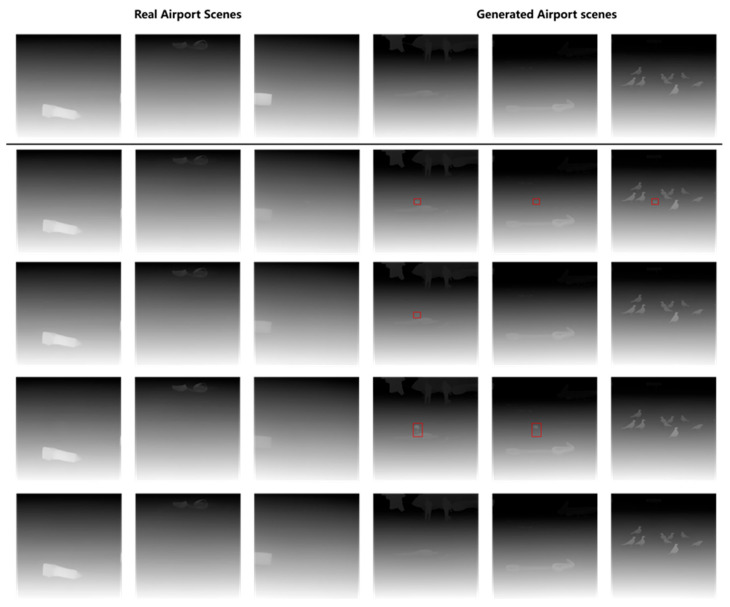
Depth estimation of real airport scenes and generated airport scenes.

Figure 14 further illustrates the depth estimation maps of the blended images, demonstrating the spatial positioning of FOD within the airport scene. Significant differences in depth information were observed in the synthetic images: when the generated FOD image was fused with a real airport scene, the depth prediction map failed to display the depth information of the FOD; however, when the generated FOD image was fused with a generated airport scene, the depth prediction map effectively reflected the depth of the FOD. This phenomenon could be attributed to several factors. Depth estimation models relied on both global and local depth cues, such as texture, shadows, object overlap relationships, and perspective information. The real airport scene image, which was a small portion extracted from a high-pole surveillance image with minimal other objects, lacked sufficient depth information, making it difficult for the model to accurately infer the depth of the newly added FOD. The complexity of the background played a significant role in depth estimation. As the real airport scene images primarily focused on the ground area and lacked prominent background objects, the model might assume that the entire image was on the same plane, thereby neglecting the depth information of the FOD. The generated airport scene images provided a more complete airport environment and contained multiple objects such as birds and airplanes, offering rich depth information that allowed the model to more easily distinguish the depth levels of the newly introduced FOD.

**Figure 15:**
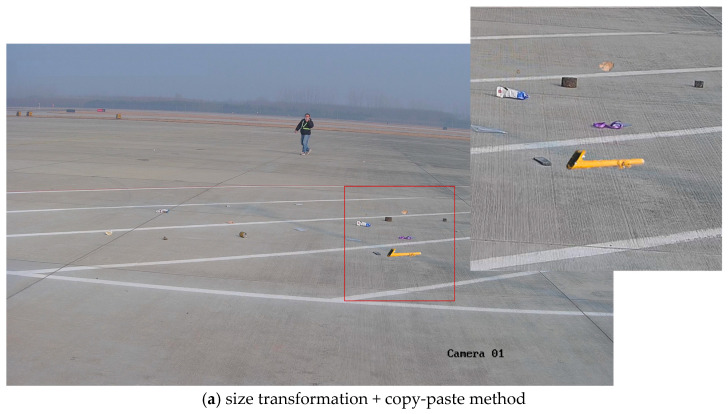

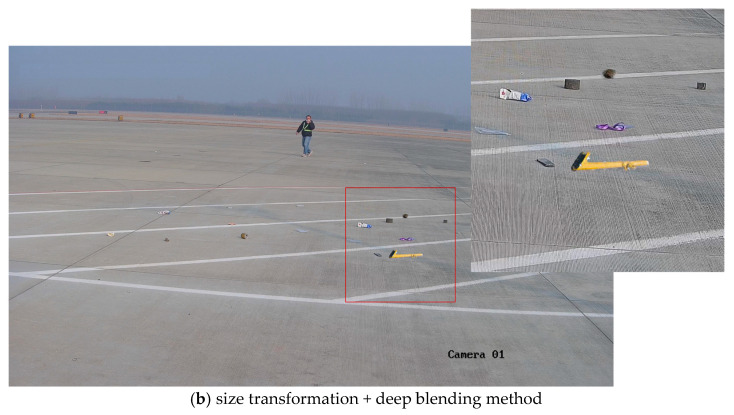
Effects of different methods on the entire image in daytime environment.

Therefore, during blending, it was best to further refine the grid to avoid the influence of other FOD in the image or to consider the fact that there was a distinct boundary between the FOD and the airport ground. When performing style transfer, only the area around the synthetic position should undergo style transformation. The images obtained through ST + DB and ST + CP were restored to real airport scenes, as shown in Figure 15. Considering the overall effect, in a good daytime environment, it was preferable to use the ST + DP method for FOD data augmentation.

#### 4.2.2. Blending Performances at Night-Time

**Figure 16:**
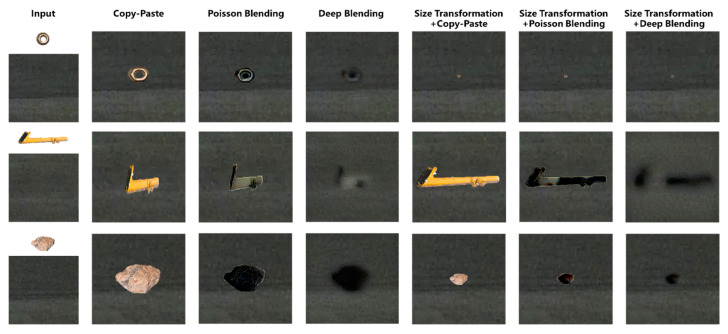
Effects of different blending methods in night-time airport environments.

**Figure 17:**
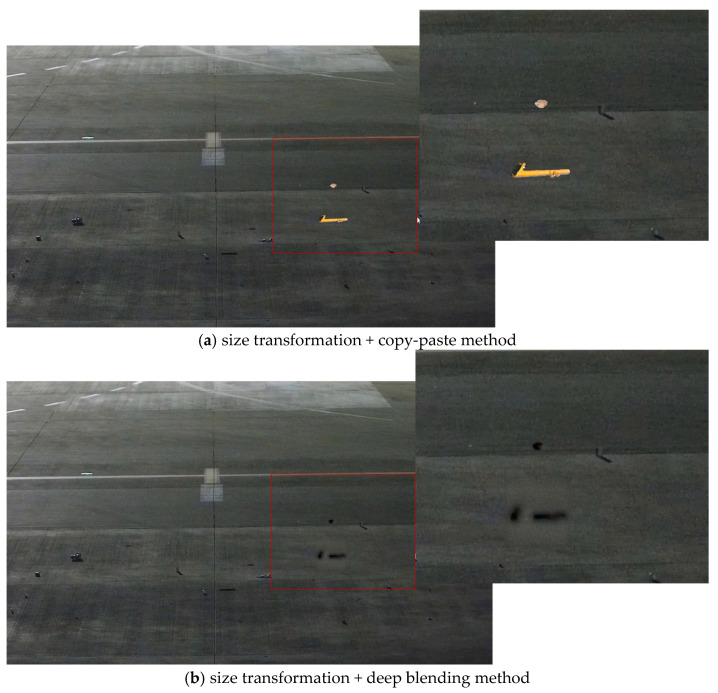
Effects of different methods on entire image in night-time environment.

As illustrated in Figure 16, a comparison of different blending methods for nuts, a pipe wrench, and a stone in a night-time apron environment was presented. Due to the low lighting conditions at night, the copy-paste method was highly conspicuous, creating a stark contrast with the surrounding environment, which rendered it unsuitable for blending in night-time scenes. In contrast, the synthetic FOD images by the Poisson method in night-time conditions showed discernible boundaries with the original images, leading to less effective integration with the night-time apron environment. On the other hand, the deep blending method allowed for a more seamless integration of FOD into the night-time scene. The images obtained through ST + DB and ST + CP were restored to real airport scenes, as shown in Figure 17. Therefore, for augmenting FOD images obtained from night-time environments, the ST + DB method was recommended for FOD data augmentation.

**Figure 18:**
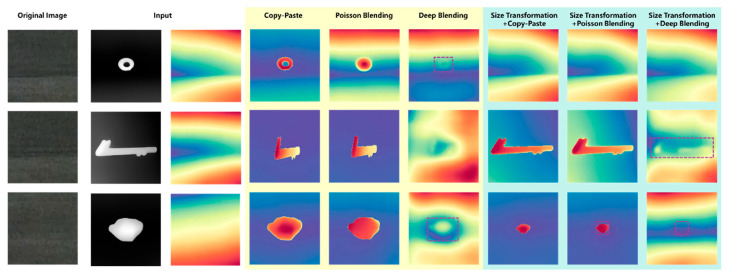
Depth estimation of different blending methods in night-time airport.

Regarding the night-time airport scenes, the performance of depth estimation varied, depending on the blending method. As shown in Figure 18, depth estimation failed to capture the positional relationship between the FOD and the airport scene when employing Poisson blending, whereas deep blending could better preserve this relationship in certain scenarios. In weak-light conditions, the overall image contrast was reduced, and Poisson blending further smoothed the gradient differences between the FOD and the background during the fusion process, leading to the loss of critical depth information. In contrast, deep blending retained the edge features of the FOD, allowing it to maintain a certain depth gradient in specific cases, thereby facilitating the depth estimation model in accurately identifying the relative position of the FOD. This indicated that deep blending was the preferable choice for FOD data augmentation in night-time airport scenes.

#### 4.2.3. Quantitative Metrics

**Figure 19:**
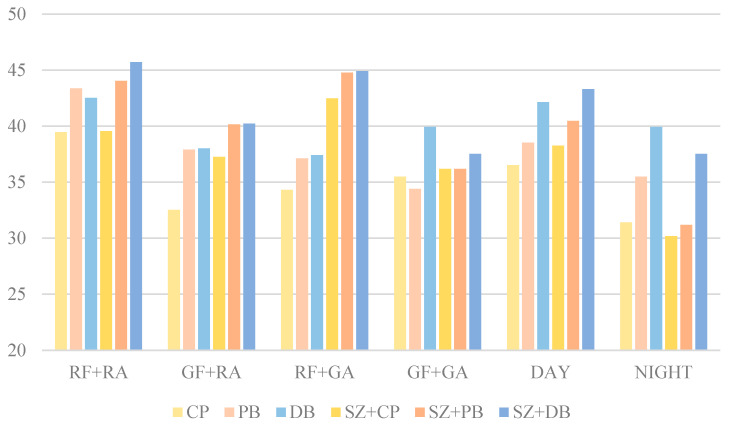
The PSNR results of different blending methods.

The PSNR results in Figure 19 illustrate the structural similarity performance of different blending methods applied to real and generated foreign-object images (RF, GF) with real and generated airport scene images (RA, GA). The PSNR values for different blending methods ranged from 30 dB to 46 dB, demonstrating significant differences in their ability to preserve image quality. Among these methods, deep blending (DB) and Poisson blending (PB) generally achieved higher PSNR values than copy and paste (CP), while the incorporation of size transformation (SZ) further improved image quality.

The CP method exhibited relatively lower PSNR values across all combinations, particularly in the GF + RA (32.53 dB) and GF + GA (35.50 dB) scenarios. The lower PSNR values could be attributed to the inability of simple copy-paste operations to ensure smooth transitions at the blending boundaries, resulting in higher errors within the blended regions. In contrast, the PB method improved PSNR values in most cases, such as RF + RA (43.36 dB) and GF + RA (37.92 dB). However, regarding the GF + GA scenario, the PSNR for PB (34.42 dB) was lower than that of the CP, suggesting that Poisson blending introduced artifacts in certain cases, leading to a decline in image quality. The DB method consistently maintained high PSNR values across different scenarios, with the highest PSNR observed in the GF + GA scenario (39.92 dB). This finding indicated that deep blending could better preserve image quality and reduce noise interference when blending generated foreign object images with generated airport scenes. Moreover, compared to the CP and PB, the DB exhibited smaller fluctuations in PSNR across different scenarios, demonstrating its stability across various blending tasks.

Incorporating size transformation significantly affected PSNR values. The results indicated that SZ + CP (38.26 dB), SZ + PB (40.46 dB), and SZ + DB (43.30 dB) achieved higher PSNR values than their respective non-size-transformed counterparts, suggesting that size transformation contributes to improved image quality. Notably, SZ + DB achieved the highest PSNR in the RF-RA (45.70 dB) and RF-GA (44.89 dB) scenarios, indicating that size transformation enhanced the structural consistency of the blended objects, while deep blending further optimized boundary smoothness, leading to superior image quality. Therefore, the SZ + DB method was recommended for practical applications, to achieve high-quality synthetic images.

Further analysis of the impact of daytime and night-time conditions on blending performance revealed that the PSNR values in the DAY category were consistently higher than those in the NIGHT category across all blending methods. For instance, under the CP method, the PSNR in the DAY scenario was 36.52 dB, whereas it dropped to 31.42 dB in the NIGHT scenario. Similarly, both the PB and DB methods exhibited PSNR values at least 3 dB higher in the DAY category than in the NIGHT category. This suggested that night-time conditions, characterized by insufficient illumination, reduced contrast and impaired the distinction between foreign objects and the background during blending.

**Figure 20:**
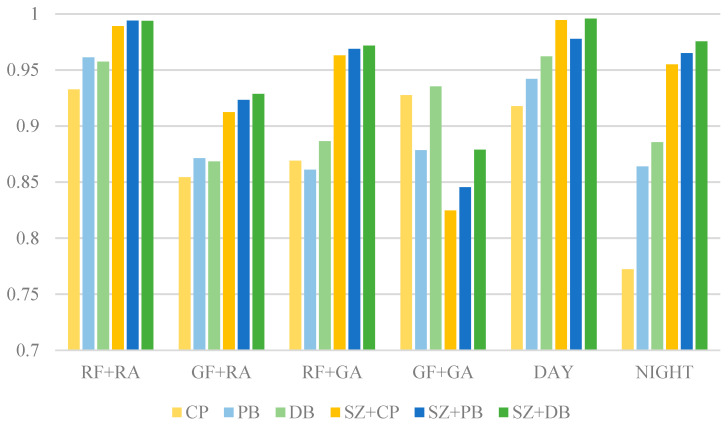
The SSIM results of different blending methods.

As shown in Figure 20, the SSIM variations for different blending methods indicated the impacts on image quality after integrating foreign-object images with airport scene images. These results were further evaluated under daytime and night-time conditions, respectively. Overall, blending involving images collected from field site (RF + RA) yielded the highest SSIM values, while the generated image blending (GF + GA) showed relatively lower SSIM scores. This finding suggested that real-image blending better preserves structural consistency, whereas synthetic image blending may introduce errors that degrade structural integrity.

The CP method performed reasonably well for real image blending (SSIM = 0.9326 in RF + RA). However, its SSIM dropped to 0.9275 in GF + GA, with a significant decline in night-time conditions (SSIM = 0.7721). This indicated that simple image pasting struggles with texture mismatches, particularly under low-light environments or when dealing with generated images.

The PB method demonstrated improved SSIM over CP in RF + RA (SSIM = 0.9611) and maintained better performance at night (SSIM = 0.8639). Its superior handling of edge transitions and illumination adjustments contributed to these results. However, in GF + GA (SSIM = 0.8784), it still exhibited some structural inconsistencies, indicating its limitations in fusing purely generated images.

The DB method maintained relatively stable SSIM values across different conditions. Its outperformed CP and PB in GF + GA (SSIM = 0.9353) and night-time scenarios (SSIM = 0.8856), suggesting its ability to effectively blend information from different sources while preserving structural integrity. Its high SSIM in daytime (SSIM = 0.9620) further confirmed its effectiveness under well-lit conditions.

Blending methods incorporating SZ (SZ + CP, SZ + PB, SZ + DB) generally achieved higher SSIM scores. In RF + RA, SZ + DB reached the highest SSIM of 0.9938, while SZ + PB also achieved a high value of 0.99401. This indicated that size adjustment contributes to improved structural consistency. However, in GF + GA, SZ + CP showed a notable drop (SSIM = 0.8247), and SZ + PB followed a similar trend (SSIM = 0.8453), suggesting that resizing introduced distortions or information loss, particularly in fully synthetic images.

All methods performed better in daytime, with SZ + DB achieving the highest SSIM (0.9957), demonstrating its effectiveness in well-lit conditions. At night, SSIM scores generally declined, with CP showing the weakest performance (SSIM = 0.7722), indicating its limitations in low-light environments. In contrast, SZ + DB retained a high SSIM of 0.9756, highlighting its robustness under night-time conditions and making it a strong choice for foreign-object data augmentation across different lighting scenarios.

In conclusion, the SZ + DB method demonstrated superior performance across both SSIM and PSNR metrics, making it the optimal choice for foreign-object data augmentation, while ensuring high image quality. In terms of SSIM, DB consistently provided stable results under various conditions, with strong adaptability in night-time environments. The incorporation of SZ further enhanced structural consistency, but introduced distortions in fully generated images. Regarding PSNR, DB exhibited greater stability compared to CP and PB, particularly in night-time scenarios where fluctuations were smaller, making it suitable for all-day data augmentation. However, the overall decline in PSNR values for night-time images indicated the necessity for specialized optimization of blending methods to enhance blending performance in low-light environments. A combination of DB and moderate SZ adjustments was recommended to achieve a balance between structural integrity and noise suppression, thereby improving the overall effectiveness of foreign-object detection tasks.

#### 4.2.4. Expert-Based Subjective Assessment

To complement traditional pixel-level metrics such as SSIM and PSNR, an expert-based subjective evaluation was conducted. This assessment was designed to measure how well the synthesized FOD images preserved spatial realism and supported accurate detection within complex airport environments.

A total of 150 blended images were selected from the augmented dataset, evenly covering three synthesis strategies: copy-paste, Poisson blending, and the proposed FOD3S method. These samples represented a variety of FOD categories, scene conditions, and object placements across different spatial regions of the scene.

An expert panel of five evaluators was assembled, including senior airport maintenance engineers, computer vision researchers, and safety inspection professionals. All evaluators assessed the images independently, and were blinded to the synthesis method used.

Each image was rated on a five-point scale across four dimensions: spatial realism (physical plausibility of the object in the scene), boundary smoothness (naturalness of edge transitions), scene consistency (alignment of lighting and texture with surroundings), and detectability (visual clarity and recognizability of the FOD). For each image, the four scores were averaged and then aggregated across the five evaluators.

The evaluation results, summarized in Table 1, show that the FOD3S framework achieved the highest subjective ratings across all four criteria. In particular, improvements in boundary integration and perceptual consistency contributed to an overall average score of 4.3, demonstrating the effectiveness of the proposed method in generating visually realistic and detection-relevant FOD imagery.

### 4.3. FOD Detection Results

#### 4.3.1. Comparison of Different Detectors with SimD

A total of 4800 high-resolution images (3200 daytime, 1600 night-time) were captured using fixed-angle cameras at three positions at airport. The dataset includes 15 categories of FOD with manual annotations. Using the proposed augmentation method, 19,200 FOD images were generated under diverse environmental conditions (rain, fog, and low-light). The synthetic-to-real ratio was set at 3:1, to ensure dataset balance. Within the dataset, 70% was used for training, 20% was used for validation, and 10% was used for test, to ensure no overlap in FOD categories or environmental conditions across splits. Two datasets were utilized in this paper, one consisting of FOD images purely from field collection, and the other one including augmented images only. Table 2 evaluates the performance of three mainstream object detectors under the application of the SimD enhancement strategy and dataset augmentation.

The introduction of SimD consistently improved mAP across all detectors, validating its effectiveness as a small-object enhancement strategy. On the original dataset, Faster R-CNN, YOLOv8, and YOLOv11 achieved absolute gains of 2.22%, 4.26%, and 1.27%, respectively, with YOLOv8 exhibiting the highest sensitivity. This disparity stemmed from the stronger reliance of single-stage YOLO-series detectors on feature alignment. By optimizing similarity distance computation, SimD enhanced small-object information in shallow features more effectively. Notably, SimD provided additional gains, even on augmented datasets (Faster R-CNN: +2.3%, YOLOv8: +2.03%, YOLOv11: +2.42%), indicating complementary mechanisms between SimD and conventional data augmentation.

To further validate the superiority of our proposed framework, we conducted an additional comparison using Poisson image blending as an alternative augmentation method. Across all detectors, the FOD3S pipeline outperformed Poisson-based augmentation. YOLOv8 achieved 80.32% mAP using FOD3S, compared to 75.45% under Poisson blending, while YOLOv11 improved from 82.24% to 83.54%, underscoring the advantages of geometry-aware and appearance-consistent synthesis. The integration of SimD yielded additional improvements over both Poisson and original augmentation setups, indicating that our similarity-aware matching strategy enhances the benefits of photometric realism with spatial consistency.

**Figure 21:**
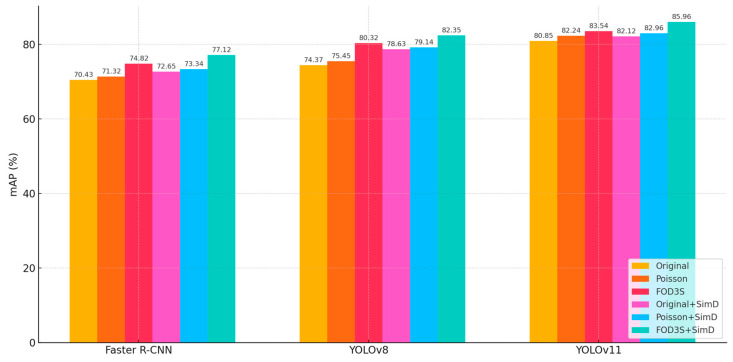
Comparison of data augmentation methods and SimD on mAP.

Performance improvements aligned with generational architectural advancements, as shown in Figure 21. YOLOv11’s baseline mAP on the original dataset (80.85%) surpassed YOLOv8 (74.37%) and Faster R-CNN (70.43%) by 6.48% and 10.42%, respectively, attributable to innovations like dynamic label assignment and cross-stage aggregation networks. However, SimD’s relative gains diminished for newer architectures (YOLOv11: +1.27% vs. YOLOv8: +4.26%), suggesting advanced models implicitly incorporated SimD’s core functionality through internal mechanisms.

Dataset augmentation substantially boosted detector performance, with YOLOv8 showing the most significant improvement (+5.95% from original to augmented dataset). Combined with SimD, a super-additive effect emerged: Faster R-CNN’s SimD gain on the augmented dataset (2.3%) exceeded its original dataset performance (2.22%), implying that expanded data diversity amplifies SimD’s utility. A plausible explanation was that augmented datasets contain richer small-object variants, enhancing the discriminative power of SimD’s metric space. YOLOv11 achieved the highest mAP (85.96%) on the augmented dataset with SimD, demonstrating that integrating traditional augmentation with targeted-feature enhancement effectively overcomes performance bottlenecks.

The experiments confirmed SimD as a robust small-object enhancement strategy, delivering consistent gains across detector architectures and synergizing effectively with data augmentation. However, performance improvements exhibited diminishing marginal returns. For YOLOv8, SimD’s gain on the augmented dataset (+2.03%) was lower than on the original dataset (+4.26%), while YOLOv11 showed a more pronounced discrepancy (augmented: +2.42% vs. original: +1.27%).

**Figure 22:**
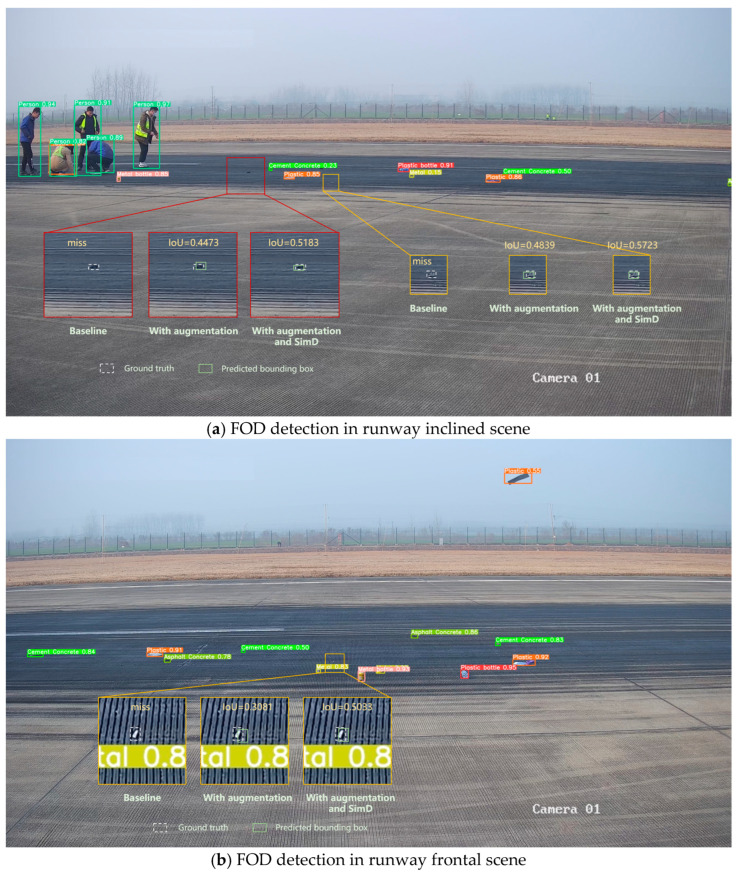

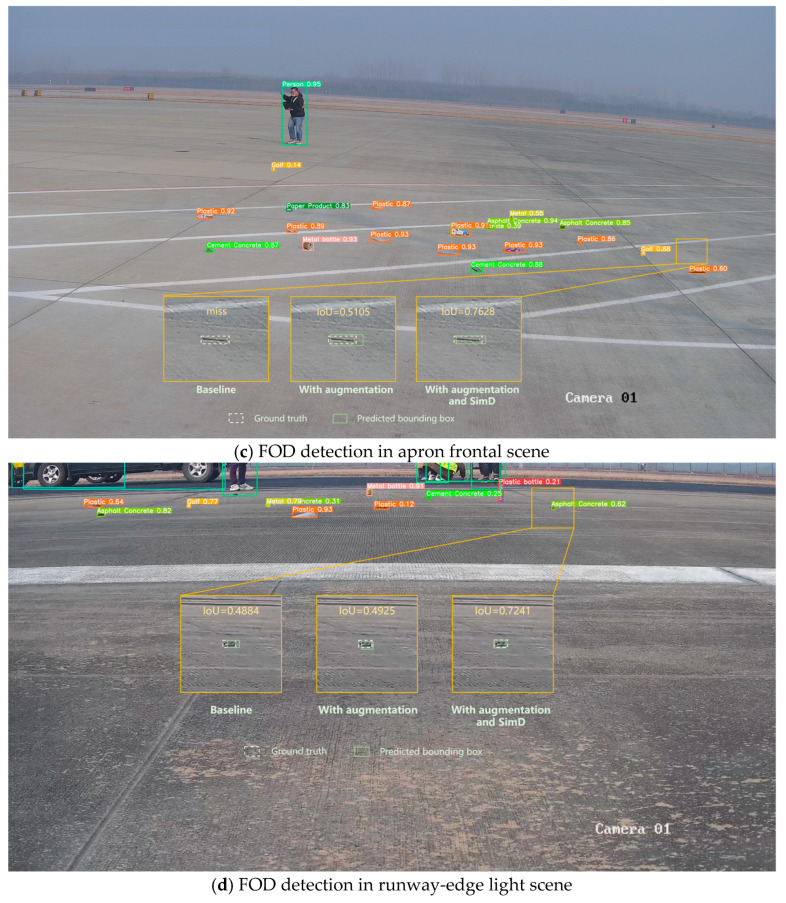
FOD detection of real airport scenes by YOLOv11.

As shown in Figure 22, the FOD detection results in real airport scenarios demonstrated that the enhanced YOLOv11 model exhibited robust performance across different airport environments. As shown in Figure 22a–c, when employing the YOLOv11 baseline for airport FOD detection, instances of missed detections were observed. Subsequent application of data augmentation successfully detected these initially missed instances, while the integration of SimD further improved the IoU metrics of predicted bounding boxes. As demonstrated in Figure 22d, the implementation of both data augmentation and SimD not only addressed detection omissions, but also enhanced the baseline model’s original prediction box IoU. These findings indicated that the synergistic combination of image enhancement strategies and the proposed detection methodology presented substantial practical value for enhancing reliability in airport FOD detection systems.

#### 4.3.2. Computational Complexity

To better evaluate the practical feasibility of deploying the proposed detection framework in real-world scenarios, the computational complexity was analyzed, together with the parameter count and inference latency of the selected object-detection models, both with and without the SimD label assignment strategy. As shown in Table 3, the comparative analysis was conducted on three representative models: Faster R-CNN, YOLOv8, and YOLOv11. The evaluation included the number of model parameters, floating-point operations (FLOPs), average inference time per image, and the achieved frame-per-second (FPS) rate on an embedded device.

Among the tested models, YOLOv11 exhibited the most favorable trade-off between detection accuracy and computational efficiency. It achieved the lowest number of FLOPs and the highest inference speed, even outperforming YOLOv8, which has a smaller parameter count but a higher computational load. This improvement is largely attributed to YOLOv11’s more optimized network architecture, including enhanced feature fusion and dynamic label assignment mechanisms. Despite its higher parameter count compared to YOLOv8, the efficient design of YOLOv11 enabled faster inference and lower resource usage.

The integration of the SimD strategy led to only a marginal increase in computational complexity across all models. Specifically, the parameter count increased by approximately 1–2%, and FLOPs increased by no more than 2.5%. However, since SimD is only active during the label assignment phase of model training and does not affect the forward inference path, the runtime cost during deployment is negligible. For example, the inference latency of YOLOv11 increased from 31.0 ms to only 32.4 ms after integrating SimD, while the FPS dropped minimally from 32.2 to 30.8, still well within real-time processing requirements.

To further validate the practical deployability of the proposed method, the YOLOv11 model enhanced with SimD was deployed on an NVIDIA Jetson AGX Orin embedded platform. Without applying any model compression techniques such as pruning or quantization, and using FP16 precision, the system achieved an average inference speed of 30.8 FPS. This result confirms that the proposed framework meets the baseline requirements for near-real-time processing in field inspection tasks. The ability to maintain high-speed inference on edge devices makes the model particularly suitable for applications such as mobile ground vehicles, low-altitude UAV patrols, or other resource-constrained scenarios in airport environments.

#### 4.3.3. Limitations and Future Work

**Figure 23:**
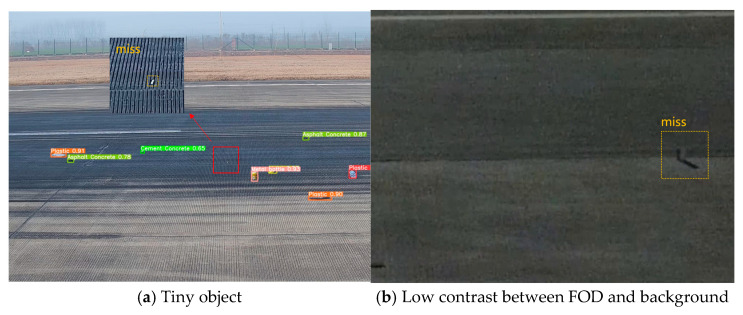
Representative failure cases of FOD detection under challenging conditions.

The representative failure cases of FOD detection under challenging conditions are presented, as illustrated in Figure 23. In Figure 23a, a metallic rod was not detected due to its circular end directly facing the camera, which significantly reduced its visible area in the image. This near-zero aspect ratio led to poor feature representation and ultimately detection failure. In Figure 23b, captured at night, a dark-colored metallic object failed to be identified because of its low contrast with the surrounding pavement. The lack of sufficient texture and luminance difference made it difficult for the model to distinguish the object from the background. These examples highlight the need for further improvement in addressing extreme viewing angles and low-visibility scenarios, such as incorporating adaptive enhancement modules or leveraging multispectral inputs.

Nevertheless, several limitations of the proposed framework should be noted. The method relies on accurate camera calibration and prior knowledge of object placement, which may be difficult to obtain or maintain in real-world dynamic airport environments. While the three-stage blending pipeline improves depth and style consistency, it may still produce imperfect results under extreme lighting, motion blur, or occlusion conditions. Moreover, although the SimD label assignment strategy enhances small-object matching, its effectiveness may degrade in highly cluttered or low-contrast scenarios. Additionally, despite demonstrating real-time inference on embedded hardware, the full augmentation pipeline remains computationally intensive and is better suited for offline dataset generation than online deployment. These limitations point toward several potential directions for future research, including improving the robustness of the blending process, accelerating the augmentation pipeline, and integrating domain adaptation mechanisms to improve transferability.

## 5. Conclusions

In this paper, we proposed Airport-FOD3S, a novel three-stage FOD synthesis framework that enables realistic, controllable image generation for airport runway inspection tasks. The pipeline integrates physical size transformation, seamless image blending, and scene-aware style transfer, allowing synthetic FOD to be projected naturally into real airport scenes while preserving spatial consistency and visual plausibility. Extensive experiments on both synthetic and real datasets demonstrate that the proposed method can significantly enhance the performance of state-of-the-art object detection models. Compared to traditional augmentation techniques such as copy-paste or Poisson blending, our FOD3S framework yields higher mAP scores on multiple detectors and shows improved generalization in cross-domain scenarios. Benefiting from its geometry-aware design, the proposed method exhibits good transferability across different airport environments. The use of perspective transformation based on spatial calibration ensures that the object placement and scaling can be adapted to various viewpoints and scene geometries. The main research findings are listed as follows:

1. A three-stage foreign-object image augmentation method was proposed. FOD was firstly transformed according to its actual sizes, was seamlessly fused with airport scene images, followed by applying style transformation, and, eventually, a comprehensive dataset for foreign-object detection was constructed;
2. The synthetic foreign object images were evaluated using DepthAnything and SSIM/PSNR metrics. The results indicated that the proposed three-stage blending method generates images with spatial distributions that were closely aligned with real-world scenarios. The SSIM and PSNR metrics outperformed other methods, reaching 0.99 and 45 dB;
3. Faster R-CNN, YOLOv8, and YOLOv11 with SimD were trained on both the original and augmented datasets. The results demonstrated that both data augmentation and SimD effectively improved foreign-object detection accuracy. Among them, YOLOv11 with SimD achieved the highest AP value of 85.96, performing well in detection tasks that match the field situation.

## Figures and Tables

**Table 1:** Expert-based subjective evaluation scores of different blending methods.

Method	Spatial Realism	Boundary Smoothness	Scene Consistency	Detectability	Average Score
Copy-Paste	2.3	2.0	2.4	2.7	2.4
Poisson Blending	3.0	2.9	3.2	3.3	3.1
FOD3S (Ours)	4.4	4.5	4.3	4.2	4.3

**Table 2:** Detection results with SimD and data augmentation.

Detector	With SimD	Original Dataset (mAP, %)	With Poisson Blending (mAP, %)	With FOD3S (Ours) (mAP, %)
Faster R-CNN	✕	70.43	71.32	74.82
	✓	72.65	73.34	77.12
YOLOv8	✕	74.37	75.45	80.32
	✓	78.63	79.14	82.35
YOLOv11	✕	80.85	82.24	83.54
	✓	82.12	82.96	85.96

**Table 3:** Detection results with SimD and data augmentation.

Detector	With SimD	Parameters (M)	FLOPs (G)	Inference Time (ms)	FPS (on Jetson Orin)
Faster R-CNN	✕	138.3	250.7	115.3	8.7
	✓	141.2	255.3	117.6	8.5
YOLOv8	✕	68.2	257.8	35.4	28.2
	✓	69.5	263.8	37.1	26.9
YOLOv11	✕	56.9	194.9	31.0	32.2
	✓	57.9	198.9	32.4	30.8

## Data Availability

Please contact the corresponding author to request access to the data mentioned in the article, but note that it cannot be used for commercial activities.
